# Clinicopathologic characteristics, outcomes, and prognostic factors of angioimmunoblastic T‐cell lymphoma in China

**DOI:** 10.1002/cam4.5248

**Published:** 2022-09-15

**Authors:** Chong Wei, Wei Li, Liping Qin, Shan Liu, Chang Xue, Kexing Ren, Zirong Zhang, Caili Liu, Fang Bao, Huilai Zhang, Hui Zhou, Zhiming Li, Huijing Wu, Liqun Zou, Lihong Liu, Hongmei Jing, Wei Zhang

**Affiliations:** ^1^ Department of Hematology, Peking Union Medical College Hospital Chinese Academy of Medical Sciences & Peking Union Medical College Beijing China; ^2^ Departments of Lymphoma Tianjin Medical University Cancer Institute and Hospital Tianjin China; ^3^ Department of Lymphoma and Hematology, Hunan Cancer Hospital/The Affiliated Cancer Hospital of Xiangya School of Medicine Central South University Changsha China; ^4^ Department of Medical Oncology Sun Yat‐sen University Cancer Center, Guangzhou, China/State Key Laboratory of Oncology in South China, Guangzhou, China/Collaborative Innovation Center for Cancer Medicine Guangzhou China; ^5^ Department of Lymphoma Medicine, Hubei Cancer Hospital, Tongji Medical College Huazhong University of Science and Technology Wuhan China; ^6^ Department of Medical Oncology, Cancer Center West China Hospital of Sichuan University Chengdu China; ^7^ Department of Hematology Fourth Hospital of Hebei Medical University Shijiazhuang China; ^8^ Peking University Third Hospital Beijing China

**Keywords:** angioimmunoblastic T‐cell lymphoma, overall survival, prognostic factor, prognostic model, treatment

## Abstract

**Background:**

This study aimed to better characterize the clinicopathologic characteristics, outcomes, and prognostic factors of AITL in China.

**Methods:**

We retrospectively analyzed 312 patients with AITL enrolled between January 2011 and December 2020 from five institutions in China.

**Results:**

The median age was 65 years, with 92.6% advanced stage, 59.7% elevated LDH, 46.1% anemia, and 44.0% hypergammaglobulinemia. The majority of patients (84.9%) received anthracycline‐based regimens with or without etoposide, and only 6.1% underwent autologous stem cell transplantation following first remission. The 5‐year OS and PFS estimates were 43.4% and 25.0% with no significant improvement of survival between patients treated during 2011–2015 and 2016–2020, respectively. Both the International Prognostic Index (IPI) and the prognostic index for PTCL, not otherwise specified (PIT), were predictive for OS. In multivariate analysis, age >70 years, elevated LDH, and albumin level <35 g/L were independent prognostic factors for OS. Combining these three factors, a novel prognostic model (the Chinese AITL score) was constructed, which stratified patients into low‐, intermediate‐, and high‐risk groups, with 5‐year OS rates of 69.0%, 41.5%, and 23.7%, respectively. This new model was successfully validated in an independent cohort.

**Conclusions:**

Patients with AITL were mainly treated with anthracycline‐based regimens, and the outcomes were still unsatisfactory in China. Our novel prognostic model may improve our ability to identify patients at different risks for alternative therapies.

## Graphical abstract

Patients with AITL in China are mainly treated with anthracycline‐based regimens with low rate of autologous stem cell transplantation and unsatisfactory outcomes. A novel prognostic model combining 3 factors (age 〉 70 years, elevated LDH, and albumin level 〈 35 g/L) was proposed which stratified patients into the low, intermediate, and high‐risk subgroups with differing outcomes.

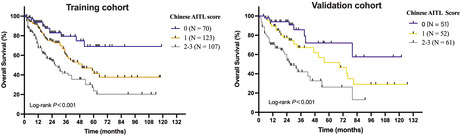

## 1 INTRODUCTION

Angioimmunoblastic T‐cell lymphoma (AITL) is a unique subtype of peripheral T‐cell lymphoma (PTCL).^1^ It was first described as a distinct clinicopathological entity in the 1970s and was classified as a new subtype of T‐cell lymphoma in the Revised European and American Classification of Lymphoid Neoplasms in 1994.^2^ AITL accounts for approximately 1% to 2% of non‐Hodgkin's lymphoma (NHL) and 18% to 36% of PTCL cases, with apparent geographical variations.^3^, ^4^, ^5^ Reliable information regarding the current prevalence of AITL in China is lacking. In a recent retrospective study of 3840 lymphoma cases, AITL accounted for 13.8% of PTCL.^6^

AITL generally occurs in elderly patients, with patients diagnosed at a median age of approximately 65 years.^7^ The clinical characteristics of AITL include generalized lymphadenopathy, hepatosplenomegaly, anemia, and polyclonal hypergammaglobulinemia. Interestingly, the disease is frequently associated with autoimmune conditions such as skin rash, arthritis, hemolytic anemia, cold agglutinins, and rheumatoid factor. Histopathologically, typical lymph nodes of AITL show complete structural effacement with marked proliferation of follicular dendritic cells and prominent branching of high endothelial venules. The neoplastic cells are typically small to medium‐sized with a clear cytoplasm. In addition to the pan T‐cell antigens, the neoplastic cells characteristically express T follicular helper (TFH) cell‐associated markers, including BCL6, CD10, CXCL13, ICOS, and PD‐1. Gene expression profiling further confirmed that CD4+ Tfh cells represent the normal counterparts of the tumor cells of AITL.^8^

AITL generally displays an aggressive clinical course and poor prognosis, with a 5‐year overall survival (OS) rate of approximately 32%–44%, reaching a plateau at approximately 6–7 years.^4^, ^5^, ^9^, ^10^, ^11^ Clinical parameters and biomarkers indicative of AITL prognosis have long been investigated. Age >60 years, high‐performance score, more than one site of extranodal involvement, mediastinal lymphadenopathy, anemia, and low platelet count have been found to be adverse prognostic factors in previous studies.^5^, ^9^, ^10^, ^11^ However, no well‐defined prognostic factors have been identified. The prognostic value of pathological factors was also poorly defined. Several prognostic models for AITL, including prognostic index for AITL, AITL prognostic index, and AITL score, have been proposed.^5^, ^10^, ^11^ However, the applicability of these novel AITL prognostic models remains unclear. Thus, this study aimed to better‐characterize the clinical characteristics and identify the prognostic factors of AITL.

## 2 PATIENTS AND METHODS

### 2.1 Study design and patients

This multicenter retrospective study evaluated two separate cohorts: a training cohort and a validation cohort. In the training cohort, a retrospective analysis was first conducted to characterize the clinicopathologic characteristics and then identify prognostic factors for proposing a new prognostic model. A total of 312 patients diagnosed with AITL between January 2011 and December 2020 at five institutions in China were retrospectively evaluated. The inclusion criteria were as the following: (1) pathologically confirmed diagnosis of AITL according to the World Health Organization (WHO) classification^12^; (2) age ≥18 years; and (3) available clinical data including baseline information for staging, treatment regimens, efficacy evaluation, and follow‐up. An independent validation cohort was enrolled to validate the results in the training cohort. Patients diagnosed within the same period and with the same inclusion criteria from another three institutions in China were included in the validation cohort. The names of the participating institutions are shown in Table S1.

This study was conducted in accordance with the Declaration of Helsinki and was approved by the institutional review board of each participating center and informed consent was waived because of the use of anonymized data.

### 2.2 Histologic and immunohistochemical analyses

At the time of enrollment, pathological specimens including slides stained with hematoxylin–eosin, immunohistochemistry, and in situ hybridization for Epstein–Barr virus encoded RNA (EBER‐ISH) were reviewed by expert hematopathologists from the pathology department of each of the eight participating centers to confirm the diagnosis. The diagnosis of AITL was based on the presence of partial or total effacement of the lymph node architecture, prominent vascularity with arborization of high endothelial venules, an extrafollicular follicular dendritic cell meshwork, an atypical population of CD4+ T cells with expression of at least two TFH markers (CD10, BCL6, PD‐1, CXCL13), and the presence of large CD20+ B immunoblasts with or without the evidence of Epstein–Barr virus (EBV) infection. The positivity of Tfh markers was defined as the expression in at least 20% of the tumor cells. Patients with concurrent/secondary diffuse large B‐cell lymphoma and nodal peripheral T‐cell lymphoma with TFH phenotype (according to the 2016 revision of the WHO classification) were excluded from analysis.

### 2.3 Clinical data collection

Clinical data were collected by case‐report forms. The clinical data included age, sex, B symptoms, immune‐related symptoms, Eastern Cooperative Oncology Group (ECOG) performance status, Ann Arbor stage, involvement of extranodal sites, International Prognostic Index (IPI) score,^13^ Prognostic Index for PTCL‐U patients (PIT) score,^14^ treatment modalities, treatment response, survival status, and the cause of death. Laboratory data recorded included baseline complete blood count, β2‐microglobulin (β2‐MG), serum lactate dehydrogenase (LDH), C‐reactive protein (CRP), immunoglobulin, and serologies for human immunodeficiency virus, hepatitis B virus (HBV), and plasma EBV viral load measured by polymerase chain reaction.

Bone marrow involvement was diagnosed based on bone marrow biopsy. Splenic involvement was supported by diffuse FDG uptake on positron emission tomography (PET), the vertical length of spleen over 13 cm, and nodular lesions or mass on computed tomography (CT) or PET/CT in our study. The involvement of other extranodal sites was determined using diagnostic tools available at diagnosis, including CT, enhanced CT, and PET‐CT. Treatment responses were evaluated according to the 2007 Revised Response Criteria for Malignant Lymphoma^15^ and 2014 Lugano classification criteria^16^ for patients diagnosed during 2010–2015 and 2015–2020, respectively. Responses were classified into complete remission (CR), partial remission (PR), stable disease (SD), or progressive disease (PD). The objective response rate (ORR) was defined as the proportion of patients who achieved PR or CR as their best response.

### 2.4 Statistical analysis

Progression‐free survival (PFS) was defined as the time from diagnosis to the first progression, relapse, or any‐cause death. Overall survival (OS) was defined as the time from diagnosis to any‐cause death or the last follow‐up. Survival analyses of PFS and OS were performed using the Kaplan–Meier method. Log‐rank test was used to compare the survival rates between the two groups. To identify the prognostic factors, univariate and multivariate analyses were performed using Cox proportional hazards regression models. Significant covariates (with *p* < 0.05) were incorporated into the multivariate analyses. Factors identified as an independent prognostic factor on OS were used to create a novel prognostic score. The performance of the new prognostic model was compared to that of the IPI and PIT scoring systems using a measure of goodness of fit (Akaike information criterion, AIC) and concordance index (Harrell C‐index), with low AIC value indicating better fit and high Harrell C‐index indicating better discrimination. Statistical analyses were performed using SPSS (version 20.0) software. Statistical significance was set at *p* < 0.05.

## 3 RESULTS

### 3.1 Patient characteristics

The median age at diagnosis was 65 years (range: 22–88 years), and the patients were predominantly male (58.3%). The patient characteristics and laboratory investigations are summarized in Table 1. Most patients (92.6%) had advanced‐stage disease. Skin rash and pleural effusion/ascites were observed in 13.1% and 14.1% of the patients, respectively. Overall, 21.5% of the patients had more than one site of extranodal involvement. Bone marrow involvement at diagnosis was observed in 18.6% of the patients. Other common sites of extranodal involvement were the spleen (32.1%), lung (6.4%), skin (6.1%), and liver (3.2%). The proportion of patients in the high‐risk group based on the IPI score (>3) and PIT score (>2) was 50/299 (16.7%) and 48/299 (16.1%), respectively.

**TABLE 1:** Clinicodemographic characteristics (*n* = 312)

Characteristic	No. (%)
Age, years	
Median (range)	65 (22–88)
>60	212 (67.9)
>70	78 (25.0)
Sex, male	182 (58.3)
ECOG performance status >1	56 (17.9)
B symptom present	150 (48.1)
Ann Arbor stage, III–IV	289 (92.6)
LDH > ULN	181/303 (59.7)
Albumin level < 35 g/L	104/305 (34.1)
IgG level > 17 g/L	33/75 (44.0)
CRP > ULN	141/187 (75.4)
β2‐MG > ULN	146/228 (64.0)
Anemia^a^	140/304 (46.1)
Platelet count <100 × 10^9^/L	37/304 (12.2)
Positive HBsAg	30/299 (9.5)
EBV‐DNA ≥500 copies/mL	66/185^b^ (35.7)
No. of extranodal sites ≥2	80/312 (25.6)
Extranodal sites	
Spleen	100 (32.1)
Lung	20 (6.4)
Skin	19 (6.1)
Liver	10 (3.2)
Bone marrow	58 (18.6)
IPI score	
0–1 (Low risk)	48/299 (16.1)
2 (Low‐intermediate risk)	109/299 (36.5)
3 (High‐intermediate risk)	82/299 (27.4)
4–5 (High risk)	50/299 (16.7)
PIT score	
0 (Group 1)	37/299 (12.4)
1 (Group 2)	115/299 (38.5)
2 (Group 3)	99/299 (33.1)
3–4 (Group 4)	48/299 (16.1)

Abbreviations: AITL, angioimmunoblastic T‐cell lymphoma; CRP, C‐reactive protein; EBV, Epstein–Barr virus; ECOG, Eastern Cooperative Oncology Group; HBsAg, hepatitis B virus surface antigen; IgG, gamma globulin; IPI, International Prognostic Index; LDH, lactate dehydrogenase; PIT, Prognostic Index for T‐cell lymphoma; ULN, upper limit of normal; β2‐MG, β2‐microglobulin.

^a^ Hemoglobin level < 120 g/L in men and 110 g/L in women.

^b^ Of the 185 cases, 61 were measured in whole blood., and the rest were measured in cell‐free plasma.

### 3.2 Pathological findings

As summarized in Table 2, immunostaining for CD3 was positive in 258/270 (95.6%) and for CD4 in 191/202 (94.6%) of the patients evaluated. For the Tfh‐associated markers, immunostaining of CD10, BCL‐6, CXCL‐13, and PD‐1 was positive in 182/269 (67.7%), 145/186 (78.0%), 141/179 (78.8%), and 155/174 (89.1%) of the patients, respectively. Background reactive CD8 + T cells were observed in 81/178 (45.5%) patients. Immunostaining for B‐cell proliferation marker CD20 and Hodgkin/Reed‐Sternberg‐like cell marker CD30 was positive in 165/290 (45.5%) and 135/210 (64.3%) of the patients, respectively. A total of 191 patients were assessed for EBV infection using EBER‐ISH. Of them, 130 (68.1%) were positive for EBER‐ISH. Among the 294 patients interpretable for the Ki‐67 index, 161/294 (57.8%), 109/294 (37.1%), and 56/294 (19.0%) had Ki‐67 index over 50%, 60%, and 70%, respectively.

**TABLE 2:** Pathological characteristics

Pathological finding	No. (%)
CD3 positive	258/270 (95.6)
CD4 positive	191/202 (94.6)
CD10 positive^a^	182/269 (67.7)
CXCL13 positive^a^	141/179 (78.8)
BCL‐6 positive^a^	145/186 (78.0)
PD‐1 positive^a^	155/174 (89.1)
Background CD8 positive	81/178 (45.5)
Background CD20 positive	165/290 (56.9)
Background CD30 positive	135/210 (64.3)
EBER‐ ISH positive	130/191 (68.1)
Ki‐67 index ≥60%	109/294 (37.1)

Abbreviation: EBER‐ISH, Epstein–Barr virus encoded small RNA in situ hybridization.

^a^ The positivity of Tfh markers was defined as expression in at least 20% of tumor cells.

### 3.3 Treatment regimens and responses

The majority of the patients (84.9%) were treated with first‐line anthracycline‐based regimens, including 181/312 (58.0%) of the patients treated with cyclophosphamide, vincristine, doxorubicin, and prednisolone (CHOP); 62/312 (19.9%) with cyclophosphamide, vincristine, doxorubicin, etoposide, and prednisolone (CHOEP); and 19 /312 (7%) with other anthracycline‐containing regimens. The remaining 13.4% of the patients received other chemotherapy regimens without anthracycline or supportive care (2.6%). Chidamide, a histone deacetylase inhibitor (HDACi), was added to chemotherapy in 72/312 (23.1%) patients. Only 19/312 (6.1%) of the patients underwent autologous stem cell transplantation (ASCT) as consolidation following the first remission. In total, 51 patients (16.3%) received maintenance therapy following remission, including chidamide maintenance in 32 patients (10.3%), thalidomide/lenalidomide maintenance in 16 (5.1%), and chidamide combined with thalidomide/lenalidomide in 3 (1.0%).

Treatment response was documented in 241 patients treated with curative intent. Of the 241 patients, 91 (37.8%) achieved CR, and another 92 (38.2%) achieved PR, yielding an ORR of 75.9%. No significant differences were observed in the CR rate (41.5% in the CHOP group [*n* = 155] vs. 33.3% in the CHOEP group [*n* = 54], *p* = 0.302) or ORR (80.0% vs. 72.2%, *p* = 0.235) between the CHOP and CHOEP groups. Similarly, adding chidamide to the CHOP/CHOEP regimen did not significantly improve the CR rate (47.9% in chidamide combined with CHOP/CHOEP [*n* = 48] vs. 36.6% in CHOP/CHOEP alone [*n* = 161], *p* = 0.160) or ORR(81.2% vs. 77.0%, *p* = 0.535). The major salvage therapies for the relapsed/refractory patients included regimens containing gemcitabine and platinum (*n* = 41), programmed cell death protein 1 (PD‐1) blockade (*n* = 14), and chidamide plus azacytidine (*n* = 8).

### 3.4 Outcomes

**FIGURE 1:**
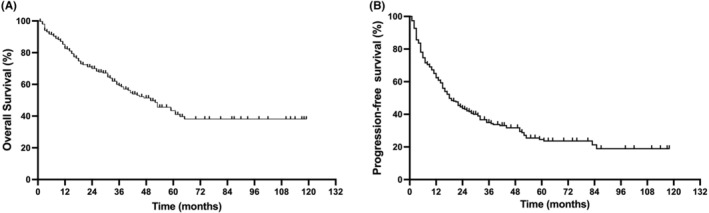
Survival of the 312 patients with angioimmunoblastic T‐cell lymphoma. Overall survival (A) and progression‐free survival (B).

**FIGURE 2:**
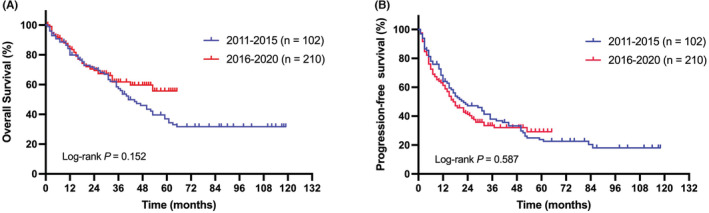
Survival at different time periods. Overall survival (A) and progression‐free survival (B).

The median follow‐up duration was 35 months (range, 2–119 months). The 1‐, 3‐, and 5‐year OS rates were 82.6%, 59.2%, and 43.4% for the entire group, with an apparent plateau at approximately 6 years (Figure 1A). The 1‐, 3‐, and 5‐year PFS rates were 62.5%, 34.9%, and 25.0%, respectively (Figure 1B). The median PFS and OS were 18 and 40 months, respectively. There were no significant differences in either PFS or OS between patients treated during 2011–2015 (n = 102) and during 2016–2020 (*n* = 210; Figure 2). However, a trend of better OS and superior plateau was observed for patients treated during 2016–2020, and this may achieve statistical significance through a longer follow‐up.

By the end of the follow‐up time, 121 deaths had been recorded, with 43.8% of deaths occurring within the first year after diagnosis and 81.8% occurring within the third year after diagnosis. The most common cause of death was disease progression (91.3%), followed by infection (7.0%).

**FIGURE 3:**
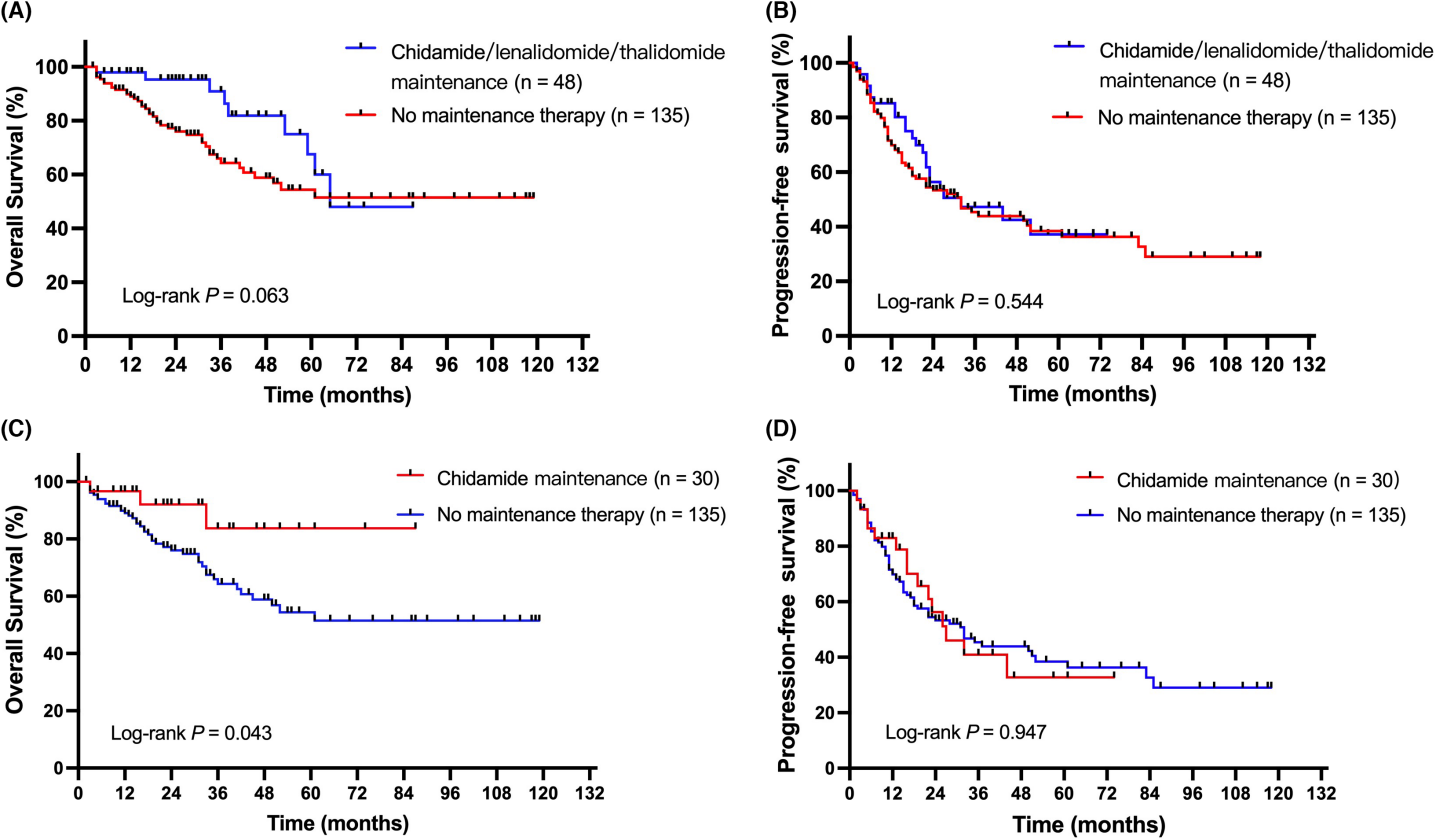
Survival of the patients with or without maintenance therapy. Overall survival (A) and progression‐free survival (B) for patients receiving chidamide, thalidomide, or lenalidomide maintenance therapy. Overall survival (C) and progression‐free survival (D) for patients receiving chidamide maintenance therapy.

To further analyze the effect of maintenance therapy, we selected patients who were treated with curative intent chemotherapy and achieved a CR or PR. A cohort of 183 patients fulfilled this criteria, of which 48 patients received chidamide, thalidomide, or lenalidomide maintenance therapy. Notably, patients who received chidamide, thalidomide, or lenalidomide maintenance therapy following the first remission tended to have better OS than patients who did not receive maintenance therapy, with 3‐year OS rates of 90.0% versus 64.3% (*p* = 0.063; Figure 3A,B). Furthermore, patients who received chidamide maintenance therapy following the first remission had significantly superior OS to patients who did not receive maintenance therapy, with 3‐year OS rates of 83.7% versus 64.3% (*p* = 0.043; Figure 3C,D). However, the PFS between patients with or without maintenance therapy were roughly the same.

### 3.5 Clinicopathologic prognostic factors

In univariate analysis, ECOG performance status ≥2, age >70 years, anemia, albumin level <35 g/L, platelet count <100 × 10^9^/L, elevated LDH, and elevated β2‐MG were adverse prognostic factors for OS. Ann Arbor stage III/IV, ECOG performance status ≥2, albumin level <35 g/L, and elevated β2‐MG levels adversely influenced the PFS (Table 3). In the multivariate analysis, only three factors were retained as independent adverse prognostic factors for OS: age > 70 years (HR = 1.94; 95% CI: 1.16–3.22; *p* = 0.011), elevated LDH (HR = 1.80; 95% CI:1.02–3.19; *p* = 0.043), and albumin level < 35 g/L (HR = 1.97; 95% CI: 1.20–3.24; *p* = 0.008).

**TABLE 3:** Univariate analysis for OS and PFS and multivariate analysis for OS

Variables	PFS			OS					
	Univariate analysis			Univariate analysis			Multivariate analysis		
	HR	95% CI	*p*	HR	95% CI	*p*	HR	95% CI	*p*
Sex, male	1.057	0.771–1.449	0.731	1.356	0.930–1.975	0.110			
Age > 70 years	1.181	0.862–1.622	0.869	**1.522**	**1.041**–**2.226**	**0.030**	**1.936**	**1.164**–**3.219**	**0.011**
ECOG performance status ≥2	**1.526**	**1.086**–**2.145**	**0.015**	**1.938**	**1.287**–**2.219**	**0.002**	1.084	0.583–2.015	0.798
Ann Arbor stage III/IV	**2.150**	**1.059**–**4.364**	**0.034**	1.829	0.747–4.479	0.186			
B symptom present	1.242	0.937–1.648	0.132	1.359	0.950–1.994	0.093			
Extranodal site >1	**1.378**	**1.003**–**1.892**	**0.048**	0.997	0.653–1.521	0.987			
Bone marrow involvement	1.264	0.869–1.838	0.221	0.797	0.463–1.373	0.414			
Anemia^a^	**1.266**	**0.946**–**1.694**	**0.011**	**2.350**	**1.641**–**3.366**	**0.000**	1.068	0.652–1.751	0.793
Platelet count <100 × 10^9^/L	1.098	0.710–1.699	0.675	1.721	**1.065**–**2.782**	**0.027**	1.594	0.849–2.995	0.147
Elevated LDH	1.268	0.939–1.711	0.121	**2.059**	**1.375**–**3.083**	**0.000**	**1.803**	**1.019**–**3.189**	**0.043**
Elevated β2‐MG	**1.666**	**1.132**–**2.451**	**0.010**	**2.360**	**1.361**–**4.092**	**0.002**	1.231	0.650–2.332	0.524
Albumin level < 35 g/L	**1.466**	**1.093**–**1.967**	**0.011**	**2.350**	**1.641**–**3.366**	**0.000**	**1.969**	**1.197**–**3.239**	**0.008**
CRP >3 mg/L	1.576	0.980–2.536	0.061	1.805	0.974–3.343	0.060			
IgG level > 17 g/L	1.053	0.601–1.844	0.857	1.730	0.863–3.468	0.122			
EBV‐DNA ≥500 copies/mL	1.343	0.925–1.952	0.121	1.470	0.927–2.330	0.101			
PD‐1 positive	1.026	0.547–1.924	0.119	0.789	0.463–1.345	0.341			
Background CD30 expression	0.891	0.621–1.276	0.527	0.891	0.568–1.388	0.618			
Background CD8 expression	0.727	0.448–1.085	0.119	0.789	0.463–1.345	0.341			
Background CD20 expression	1.243	0.924–1.670	0.149	1.076	0.743–1.559	0.698			
EBER‐ ISH positive	1.441	0.938–2.215	0.095	1.091	0.613–1.942	0.768			
Ki‐67 index >60%	1.208	0.894–1.633	0.218	**1.638**	**1.129**–**2.347**	**0.009**	1.256	0.781–2.021	0.348

Factors with *p* < 0.05 were shown in bold value.

Abbreviations: CRP, C‐reactive protein; EBV, Epstein–Barr virus; ECOG, Eastern Cooperative Oncology Group; IgG, gamma globulin; LDH, lactate dehydrogenase; β2‐MG, β2‐microglobulin.

^a^ Hemoglobin <120 g/L in men and 110 g/L in women.

We also evaluated pathological features as possible prognostic factors, including PD‐1 expression, background CD8, CD20, CD30 expression, EBER‐ISH, and Ki‐67 index. In univariate analysis, only a Ki‐67 index >60% (HR = 1.64; 95% CI: 1.13–2.35; *p* = 0.009) significantly influenced OS. However, in multivariate analysis, the Ki‐67 index was not retained as an independent prognostic factor for OS.

### 3.6 Prognostic model for AITL

**FIGURE 4:**
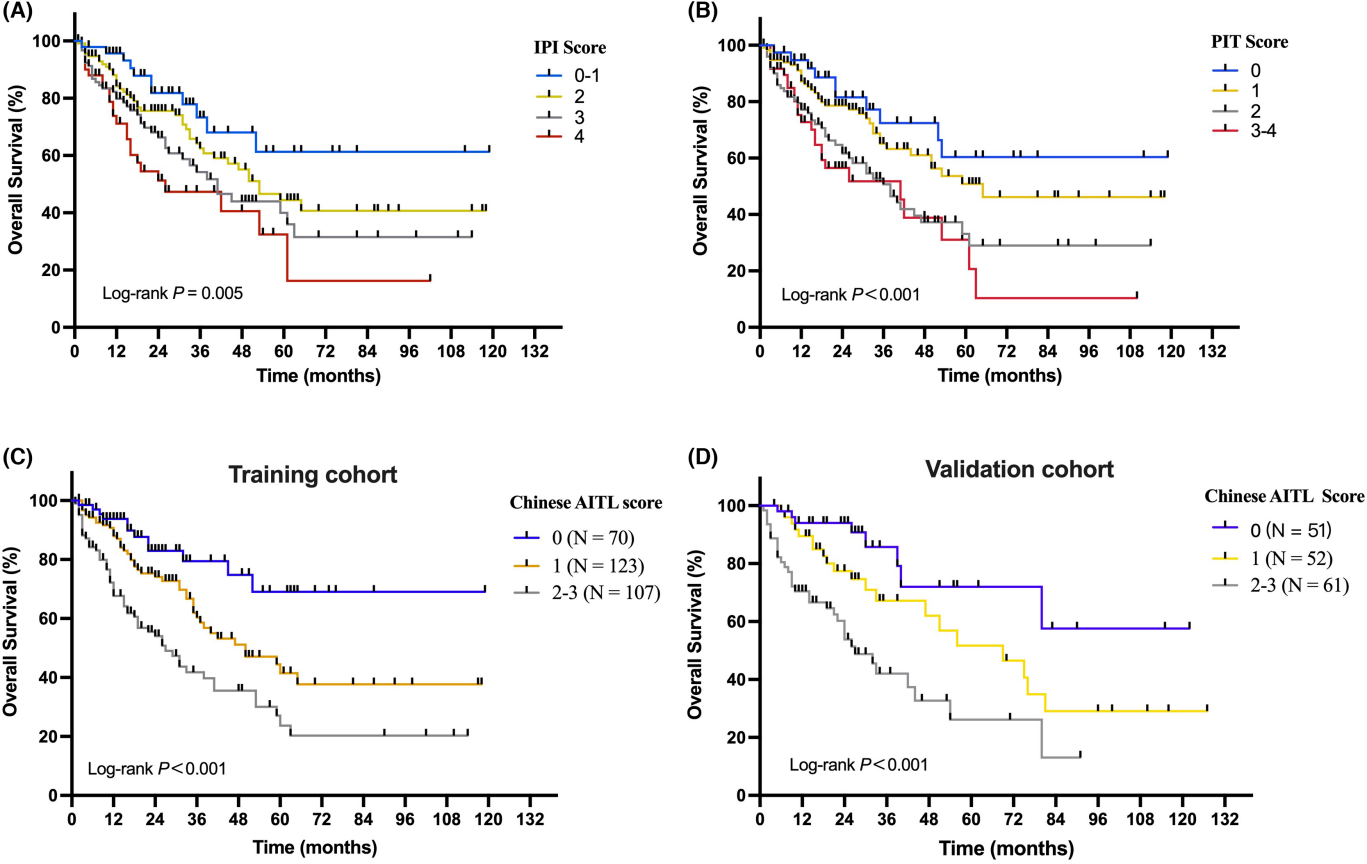
Overall survival of the patients according to different prognostic models. International Prognostic Index (A). Prognostic Index for Peripheral T‐Cell Lymphoma, Unspecified (B). Chinese AITL prognostic model (C). Overall survival for validation cohort using the Chinese AITL prognostic model (D).

The prognostic values of the PIT and IPI scoring systems were evaluated in this study. Both PIT and IPI were predictive of OS. The 5‐year OS rates were significantly different according to the IPI scores (low risk: 61.2% vs. low‐intermediate risk: 44.4% vs. intermediate‐high risk: 40.0% vs. high‐risk: 32.4%, *p* = 0.005) and the PIT scores (group 1: 60.3% vs. group 2: 50.8% vs. group 3: 33.1% vs. group 4: 31.1%, *p* < 0.001), as shown in Figures 4A,B.

Combining the three factors identified in the multivariate analysis, we constructed a new prognostic model, the Chinese AITL score. Patients were stratified into three risk groups: low‐risk group, 0 adverse factors; intermediate‐risk group, 1 factor; and high‐risk group, 2–3 adverse factors. The median OS was not reached in the low‐risk group, 50 months in the intermediate‐risk group, and 27 months in the high‐risk group (Figure 4C). The 5‐year OS rates were 69.0%, 41.5%, and 23.7% for patients in the low‐, intermediate‐, and high‐risk groups, respectively (*p* < 0.001). Among the IPI, PIT, and the Chinese AITL scoring systems, the Chinese AITL scoring system demonstrated the greatest discriminant power, with the highest Harrell C‐index (0.6585 vs. 0.608 for IPI score and 0.610 for PIT score) and lowest AIC (1152 vs. 1159 for IPI score and 1157 for PIT score).

The Chinese AITL prognostic model was tested in an independent cohort of patients with AITL for validation. A total of 164 patients from three other centers were evaluated using the Chinese AITL prognostic model. Significant survival differences in OS were maintained between the low‐ and high‐risk groups (*p* < 0.001) in this validation cohort (Figure 4D). The median OS was not reached in the low‐risk group, 69 months in the intermediate‐risk group, and 27 months in the high‐risk group. The 5‐year OS rate in the low‐, intermediate‐, and high‐risk groups was 72.0%, 51.7%, and 26.2%, respectively.

## 4 DISCUSSION

This retrospective study included patients with AITL from five large centers across north and south China for a period of 10 years. In keeping with previous studies, we confirmed that AITL is a disease of elderly patients and typically present with features of poor prognostic factors for B‐cell NHL: 67.9% of the patients were older than 60 years: 92.6% presented with Ann Arbor stages III–IV disease; 59.7% had elevated LDH level; and 25.6% had more than one site of extranodal involvement, with the spleen, liver, lung, and skin being the most common extranodal sites. Notably, skin rash (13.1%), pleural effusion/ascites (14.1%), and a positive Coombs test (44.7%) appeared to be distinctive autoimmune manifestations of AITL. To our best knowledge, this analysis represents the largest AITL cohort reported to date, allowing for a real‐world assessment of clinicopathological features, treatment outcomes, survivals, and prognostic factors for patients treated in the contemporary era in China.

Most patients (84.9%) were treated with CHOP‐like regimens, and a small proportion received etoposide (19.9%), which may partly reflect the older age of our cohort. In previous studies, adding etoposide to CHOP was reported to improve the ORR and event‐free survival, but not OS, and only for younger patients with AITL (age ≤ 60 years).^17^, ^18^ In our study, 55.7% of the patients treated with CHOEP were aged >60 years. Consistent with previous studies, no beneficial effect of etoposide addition was observed in either the CR rate or ORR when older patients were included. In recent years, HDACis such as belinostat, romidepsin, and chidamide, have been approved for the treatment of relapsed/refractory PTCL. Chidamide is a novel HDACi independently developed in China. The efficacy and safety of HDACis combined with CHOP/CHOEP regimens have been assessed in several clinical trials. In a randomized phase III study evaluating romidepsin plus CHOP versus CHOP in patients with untreated PTCLs, the addition of romidepsin did not improve PFS, response rate, or OS.^19^ In a phase Ib/II study evaluating chidamide plus CHOEP in patients with untreated PTCL, modest efficacy was reported, with a CR rate of 40.7% and ORR of 60.2%, showing no clear benefit of adding chidamide to CHOEP.^20^ Similar to previous studies, the addition of chidamide to the CHOP/CHOEP regimen did not significantly improve the response rate in our study.

Upfront ASCT is recommended to improve the prognosis of PTCLs. In the Nordic Lymphoma Group (NLG T‐01) study, the largest prospective trial evaluating ASCT consolidation, and the 5‐year PFS was 44% in patients who were consolidated with ASCT.^21^ Cumulative evidence from other studies, including a large population‐based study from the Swedish Lymphoma Registry and a prospective study from the United States COMPLETE registry, further supports upfront ASCT consolidation for eligible patients with AITL.^17^, ^22^ However, the benefits of upfront ASCT have not been investigated in randomized studies. In our study, only a small proportion of patients (6.1%) underwent consolidative ASCT after the first remission. The low percentage of ASCT may reflect the older age of our cohort, as well as the geographic variations in economic state and clinical practice patterns. Promoting upfront ASCT consolidation following first remission may be an effective strategy to improve the long‐term survival of patients with AITL in China.

Although the majority of patients with AITL are sensitive to chemotherapy, the response duration is often short, with relapse frequently that result in poor survival. In our study, responding patients (CR or PR response) who received chidamide maintenance therapy showed significantly superior OS and similar PFS than patients who did not receive maintenance therapy. The superiority of OS on the basis of similar PFS of the maintenance group indicates that chidamide maintenance may prolong the survival with tumor, slow the progression of the disease, and result in more opportunities for further treatment. In addition, chidamide is an oral tablet with the advantage of being convenient to use as long‐term maintenance. Previously, long‐term treatment with romidepsin and pralatrexate was only reported in case reports.^23^, ^24^ To the best of our knowledge, this study is the first to evaluate the importance of HDACi used as maintenance therapy in patients with AITL. The benefits of chidamide maintenance should be further evaluated in prospective randomized trials.

Overall, the survival of patients with AITL treated with standard chemotherapy regimens was disappointing, with 5‐year OS and PFS estimates of 43.4% and 25.0%, respectively. These outcomes fall within the range of those reported in previous large population‐based studies (5‐year OS rates of 32%–44% and 5‐year PFS rates of 18%–33%).^5^, ^9^, ^10^, ^11^, ^17^ Another study utilizing the Surveillance, Epidemiology, and End Results (SEER) database that evaluated a cohort of 1207 patients with AITL reported no survival differences among groups diagnosed in the five time periods (1992–1998, 1999–2001, 2002–2004, 2005–2007, and 2008–2010).^25^ Our study yielded equally disappointing results, and no significant differences of survival were observed between patients treated during 2011–2015 and during 2016–2020. However, a trend of better OS and superior plateau was observed for patients treated during 2016–2020. The reasons for this may include the following. First, the increasing use of novel therapies including HDACi, demethylating agents, PD‐1 blockade, and brentuximab vedotin as salvage treatment may offer more options for the relapsed/refractory patients. Second, chidamide, thalidomide, or lenalidomide maintenance therapy may also contribute to the superior plateau of the OS curve.

To date, only a few studies have aimed to identify prognostic factors of AITL, yielding controversial results. However, no well‐defined clinical or pathological prognostic factors have been identified. A study from the International Peripheral T‐cell Lymphoma Project suggested that age >60 years, performance score ≥2, more than one site of extranodal involvement, presence of B symptoms, and platelet <150 × 10^9^/L were predictive of poor prognosis.^11^ Recently, Ranjana et al identified four adverse prognostic factors of AITL: ECOG performance status >2, age >60 years, elevated CRP, and elevated β2‐MG.^5^ In the current study, age >70 years, elevated LDH level, and albumin level <35 g/L were identified as independent adverse prognostic factors for OS. The association between EBV infection status and prognosis in patients with AITL remains controversial. Positive EBER‐ISH status was reported to be associated with significantly better PFS than EBER‐negative status among younger patients with AITL.^26^ In another study from the GELA trial, a high EBV viral load at diagnosis (EBV DNA >100 copies/μg) was associated with shorter PFS.^27^ Conversely, neither whole blood EBV‐DNA ≥500 copies/mL nor positive EBER‐ISH in the tumor tissue was predictive of poor PFS and OS in our study.

Few studies have attempted to identify pathologic prognostic factors of AITL, and none of them have proven clinical value. Our study specifically investigated the potential prognostic value of PD‐1 expression, background CD8, CD20, CD30 expression, and Ki‐67 index. In 2006, Went et al. evaluated the expression of 19 markers using a tissue microarray immunohistochemical analysis in a cohort of patients with AITL and PTCL‐NOS. They proposed a modified PIT model that incorporated a Ki‐67 index ≥80%.^28^ In this respect, the significance of the Ki‐67 index was also evaluated in our study, but it failed to show independent prognostic value for OS in multivariate analysis. In a study evaluating the correlation between gene signatures and clinical outcomes in AITL, the B‐cell signature was associated with favorable outcomes, whereas the cytotoxic signature of CD8+ T cells was associated with poorer outcomes.^29^ However, immunohistochemistry of both the T‐cell marker CD8 and background B‐cell marker CD20 failed to show a significant influence on survival.

As shown in Table 4, different prognostic models have been proposed for PTCLs, including the AITL. In our study, a novel prognostic model was constructed for AITL using the three prognostic factors identified in the multivariate analysis for OS, including age >70 years, elevated LDH level, and albumin level <35 g/L, assigning 1 point for each factor. Patients with AITL were stratified into the low‐risk (0 points), intermediate‐risk (1 point), and high‐risk (2–3 points) subgroups, with 5‐year OS rates of 69.0%, 41.5%, and 23.7%, respectively. The abovementioned three factors are quite common and practical in clinical use. Our model demonstrated greater discriminant power than the IPI and PIT and was successfully validated in an independent cohort.

**TABLE 4:** Prognostic factors and models of AITL

Prognostic mode	No. of cases	Nature of the study	Enrollment time period	Factors	Risk groups	5‐year OS rate (%)
Prognostic Index for Peripheral T cell Lymphoma (PIT)^14^	385 (PTCLs)	Retrospective	1989–2001	Age >60 years; ECOG PS ≥2; elevated LDH; bone marrow involvement	Group 1 (0) Group 2 (1) Group 3 (2) Group 4 (3–4)	62.3 52.9 32.9 18.3
Prognostic Index for AITL (PIAI)^11^	243 (AITL)	Prospective	1990–2002	Age >60 years; ECOG PS ≥2; >1 extranodal site; presence of B symptoms; platelet count <150 × 10^9^/L	Low risk (0–1) High risk (2–5)	44 24
Tokunaga et al.^10^	207 (AITL)	Retrospective	1990–2008	Age >60 years; elevated WBC; elevated IgA; anemia; platelet count <150 × 10^9^/L; >1 extranodal sites	Low risk (0–1) Low‐intermediate risk (2) High‐intermediate risk (3) High risk (4–6)	85^a^ 62^a^ 51^a^ 12^a^
Eladl et al.^26^	270 (AITL)	Retrospective	1990–2016	EBER negative status; thrombocytopenia; elevated IgA	Low risk (0–1) High risk (2–3)	91^a^ 18^a^
AITL score^5^	282 (AITL)	Prospective	2006–2018	Age >60 years; ECOG PS >2; elevated CRP; elevated β2‐MG	Low risk (0–1) Intermediate risk (2) High risk (3–4)	63 54 21

Abbreviations: AITL, angioimmunoblastic T‐cell lymphoma; CRP, C‐reactive protein; EBER, Epstein–Barr virus‐encoded small RNA; ECOG, Eastern Cooperative Oncology Group; LDH, lactate dehydrogenase; PS, performance status; PTCL, peripheral T‐cell lymphoma; WBC, white blood cell; β2‐MG, β2‐microglobulin.

a 3‐year OS rate.

Although this study provided novel information regarding AITL, it also has some limitations. The most important limitations of our study was the lack of a centralized pathologic review. The antibodies utilized and the interpretations of immunohistochemical stains may vary in different participating centers. Second, the possibility of unrecognized biases could not be ruled out owing to the retrospective nature of the study. Third, genetic alterations and expression patterns were not included in this study. A better prognostic model may rely on the inclusion of genetic factors in addition to immunohistochemical features and clinical factors.

In conclusion, patients with AITL are mainly treated with anthracycline‐based regimens, and the outcomes are still unsatisfactory. Age >70 years, elevated LDH level, and albumin level <35 g/L at the initial diagnosis are independent prognostic factors for OS. Our novel prognostic model combining the abovementioned three factors may improve the risk stratification for AITL.

## AUTHOR CONTRIBUTIONS

**Wei Chong:** Conceptualization (equal); data curation (equal); formal analysis (equal); investigation (equal); writing – original draft (equal); writing – review and editing (equal). **Wei Li:** Data curation (supporting); investigation (supporting). **Liping Tan:** Data curation (supporting); investigation (supporting). **Shan Liu:** Data curation (supporting). **Chang Xue:** Data curation (supporting). **Kexin Ren:** Data curation (supporting). **Zirong Zhang:** Data curation (supporting). **Caili Liu:** Data curation (supporting). **Fang Bao:** Data curation (supporting). **Hui Lai Zhang:** Conceptualization (supporting); formal analysis (supporting); investigation (supporting). **Hui Zhou:** Conceptualization (supporting); formal analysis (supporting); methodology (supporting). **Zhi‐Ming Li:** Conceptualization (supporting); formal analysis (supporting); methodology (supporting). **Huijing Wu:** Conceptualization (supporting); formal analysis (supporting); methodology (supporting). **Liqun Zou:** Conceptualization (supporting); formal analysis (supporting); methodology (supporting). **Lihong Liu:** Conceptualization (supporting); formal analysis (supporting); methodology (supporting). **Hongmei Jing:** Conceptualization (supporting); formal analysis (supporting); methodology (supporting). **Wei Zhang:** Conceptualization (equal); data curation (equal); formal analysis (equal); investigation (equal); methodology (equal); writing – review and editing (equal).

## FUNDING INFORMATION

National Natural Science Foundation of China (Nos. 81872902 and 82073917). Natural Science Foundation of Beijing Municipality (No. 7202154).

## CONFLICT OF INTEREST

The authors declare no potential conflicts of interest.

## ETHICS APPROVAL AND CONSENT TO PARTICIPATE

The study was conducted in accordance with the Declaration of Helsinki and was approved by the institutional review board of each participating center. The requirement for informed consent was waived because of the use of anonymized data.

## Supporting information


Table S1

## Data Availability

The data generated in this study are available upon request from the corresponding author.
