# Supplementary material for: Clinicopathologic characteristics, outcomes, and prognostic factors of angioimmunoblastic T‐cell lymphoma in China
Source: Cancer Med. 2022 Sep 15;12(4):3987–98. doi: 10.1002/cam4.5248 (PMC9972121; doi:10.1002/cam4.5248)
Supplement: Supplementary file 1 — Table S1 [file CAM4-12-3987-s001.docx]

**Table S1.** The names of the participating institutions

| **Training centers** | **﻿Validation centers** |
| --- | --- |
| 1. ﻿Peking Union Medical College Hospital  2. Tianjin Medical University Cancer Institute and Hospital  3. Hubei Cancer Hospital  ﻿4. Hunan Cancer Hospital/The Affiliated Cancer Hospital of Xiangya School of Medicine  ﻿5. Sun Yat-Sen University Cancer Center | ﻿6. Peking University Third Hospital  ﻿7. West China Hospital of Sichuan University  8. Fourth Hospital of Hebei Medical University |
